# Length of Menstrual Cycle and Risk of Endometriosis: A Meta-Analysis of 11 Case–Control Studies

**DOI:** 10.1097/MD.0000000000002922

**Published:** 2016-03-07

**Authors:** Ming Wei, Yanfei Cheng, Huaien Bu, Ye Zhao, Wenli Zhao

**Affiliations:** From the Departments of Obstetrics and Gynecology (MW), Clinical Research (YZ), and Neurology (WZ), Nankai Hospital, Tianjin Academy of Integrative Medicine; and Department of Public Health, School of Chinese Medicine (HEB) and Graduate School (YFC, WLZ), Tianjin University of Traditional Chinese Medicine, Tianjin, China.

## Abstract

Endometriosis is a complex disease that affects a large number of women worldwide and may cause pain and infertility.

To systematically review published studies evaluating the relationship between menstrual cycle length and risk of endometriosis.

We searched the Cochrane Library, PubMed, Web of Science, and EMBASE in databases in July 2014 using the keywords “case–control studies,” “epidemiologic determinants,” “risk factors,” “menstrual cycle,” “menstrual length,” “menstrual character,” and “endometriosis.”

We included case–control studies published in English that investigated cases of surgically confirmed endometriosis and examined the relationship between endometriosis risk and menstrual cycle.

Eleven articles that met the inclusion criteria included data of 3392 women with endometriosis and 5006 controls. Fixed-effects and random-effects models were used for the evaluation.

For the association of risk of endometriosis and menstrual cycle length shorter than or equal to 27 days (SEQ27) or length longer than or equal to 29 days (LEQ29), the odds ratio was 1.22 (95% confidence interval [CI]: 1.05–1.43) and 0.68 (95% CI: 0.48–0.96), respectively.

In conclusion, this meta-analysis suggests that menstrual cycle length SEQ27 increase the risk of endometriosis and cycle length LEQ29 decrease the risk.

## INTRODUCTION

Endometriosis is a complex gynecologic disorder characterized by benign proliferation of ectopic endometrial glands and stroma in the peritoneal cavity, with dysmenorrhea, pelvic pain, and infertility being typical symptoms.^1,2^ It affects approximately 10% to 15% of women of reproductive age and as many as 50% of women with infertility.^3^

Genetic predisposition and environmental factors are considered to be closely associated with the development of endometriosis.^4–7^ However, the pathogenesis of this disease is currently not very clear. Three main theories used to explain the manifestations and invasion are the retrograde menstruation theory, Müllerian metaplasia theory, and lymphatic spread theory.^8^

Many studies have surveyed the risk factors associated with the disease. It has been shown that age, race, body mass index, alcohol usage, cigarette smoking, and menstrual characters (such as the length and regularity of menstrual cycles, intensity of menstrual flow, and dysmenorrhea) are associated with the incidence of endometriosis.^9–12^

Menstrual cycle characteristics affecting the frequency of exposure to menstruation may be related to the incidence of endometriosis.^13^ Thus, several observational studies and a meta-analysis have provided some evidence that early age at menarche increases the risk of endometriosis.^14,15^ The length of the menstrual cycle varies greatly among women (ranging from 21 to 35 days), with 28 days designated as the average length.^16^ Accordingly, 28 days was used as the cut-off value for group assignment in a number of studies relevant to endometriosis.^17–22^ We placed demarcation points at 27 and 29 days and defined menstrual cycle length shorter than or equal to 27 days as SEQ27 and longer than or equal to 29 days as LEQ29. The majority of observational studies have examined the relationship between menstrual cycle length, 1 of the menstrual characteristics, and endometriosis, and some have found an association between a shorter menstrual cycle and the incidence of this disease.^20–23^ On the contrary, Sangi-Haghpeykar's study^24^ showed that women with long cycle lengths were 1.8 times more likely to develop endometriosis than those with short cycle lengths. Dramatically, some studies^11,23,25^ did not find any significant correlation between short menstrual cycle length and endometriosis at all. Therefore, the association between menstrual cycle length and endometriosis needs further confirmed. The aim of the present study is to perform a meta-analysis to examine the association between menstrual cycle length and endometriosis.

## METHODS

### Search Strategy

A systematic search was performed in the Cochrane Library, PubMed, MEDLINE, and EMBASE databases for all case–control studies published between January 1993 and July 2014 using the following keywords: “Case–control studies,” “epidemiologic determinants,” “risk factors,” “menstrual cycle,” “menstrual length,” “menstrual character,” and “endometriosis.” Reference lists of selected publications were then searched manually by 1 of the authors to identify additional relevant studies. Two authors reviewed the papers and independently selected eligible articles for the systematic review.

### Study Selection

Studies were included if they investigated cases of surgically confirmed endometriosis; examined the relationship between endometriosis risk and menstrual cycle; were case–control studies; and were full-length articles published in English. Only original publications were included; conference abstracts and dissertations were excluded.

### Data Extraction and Quality Assessment

Data were extracted independently by 2 investigators, and discrepancies were resolved by discussion. The following details were collected: first author's last name, year of publication, country of origin, study design, number of subjects, age, length of menstrual cycle in days, and odds ratios (ORs) of endometriosis and corresponding confidence intervals (CIs). The quality of primary studies was evaluated using the Newcastle–Ottawa Scale (NOS) according to 3 criteria: participant selection, comparability of study groups, and assessment of exposure.^26^

### Statistic Analysis

ORs and 95% CIs for menstrual cycle length and endometriosis were calculated in a random-effects model and a fixed-effects model when appropriate. Both the Cochran Q statistic to test for heterogeneity and the I^2^ statistic to quantify the proportion of the total variation due to heterogeneity were calculated. A *P* value of more than the nominal level of 0.10 for the Q statistic indicated a lack of heterogeneity across studies, allowing for the use of a fixed-effects model; otherwise, the random-effects model was used. To explore sources of heterogeneity across studies, subgroup analysis was performed. Sensitivity analysis was performed to assess the stability of results. All statistical analyses were performed with the RevMan 5.3 software.

## RESULTS

### Results of the Literature Search

**FIGURE 1:**
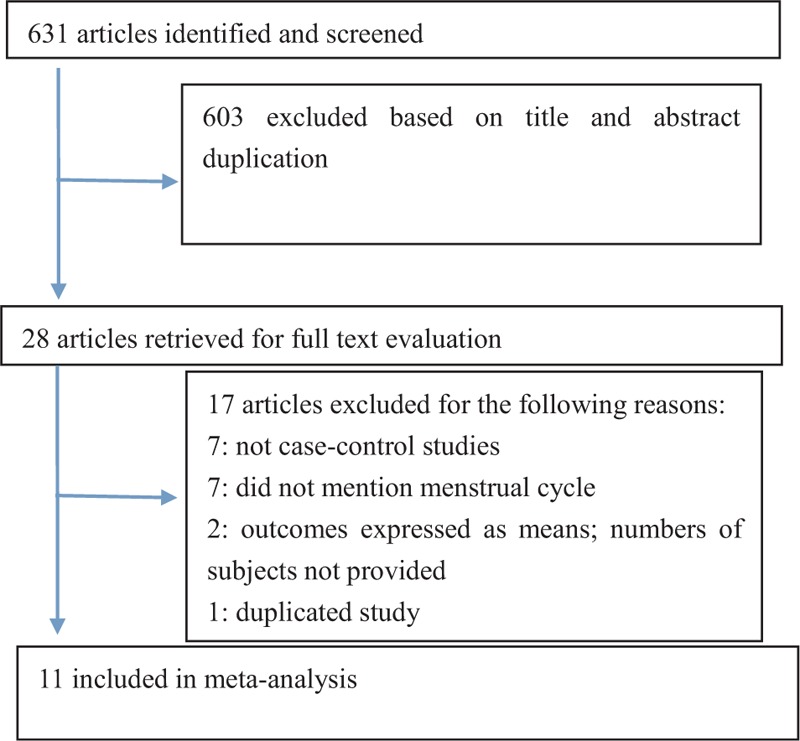
Flow diagram illustrating the study selection process.

Of the 631 reports initially identified from the database search, 603 articles were excluded after carefully reviewing the titles and abstracts because of low relevance to the present study (Figure 1). Among the 28 remaining articles, 17 more were excluded after an in-depth examination for the following reasons: 7 were not case–control studies, 7 did not mention menstrual cycle, 2 only presented mean values and not numbers of subjects, and 1 had similarities with 1 of the selected reports. Thus, only 11 studies fulfilled the predefined entry criteria and were ultimately selected for meta-analysis.

### Study Characteristics

Table 1 summarizes the 11 case–control studies selected for meta-analysis. Numbers of subjects in the studies published between 1993 and 2014 ranged from 186 to 2777, and there were 3392 cases of surgically diagnosed endometriosis and 5006 controls (Table 1).^18–22,24,25,27–30^ Fortunately, we received the original data from one author of study^22^ and used them in data analysis. The studies were conducted in Italy (n = 4), the United States (n = 2), Canada (n = 2), Australia (n = 1), Malaysia (n = 1), and Iran (n = 1). Subjects were generally younger than 55 years,^18–21,28,29^ although women up to 64 years old were included in 1 study.^24^ Four studies^22,25,27,30^ did not provide the subjects’ age explicitly but used age ranges. Reports differed with respect to the description of menstrual cycle. Five studies described menstrual cycle as “regular” or “irregular,”^19,22,27,29,30^ and 2 of them did not mention the duration.^29,30^ Nine studies^18–22,24,25,27,28^ used clearly defined intervals for the menstrual cycle length but the intervals in different studies were not the same. Five studies^18–20,22,25^ provided the numbers of women in case and control with the length SEQ27 and 5 studies^19,20,22,24,28^ provided the cases numbers with the length LEQ29. Three studies used age-matched controls.^21,28,30^ In particular, 1 study^28^ utilized age-matched twins as case and control subjects, whereas another^18^ employed 2 control groups: 100 controls who were friends of the patients with endometriosis and 98 medical controls (patients from the same medical practice with conditions other than endometriosis).

**TABLE 1:**
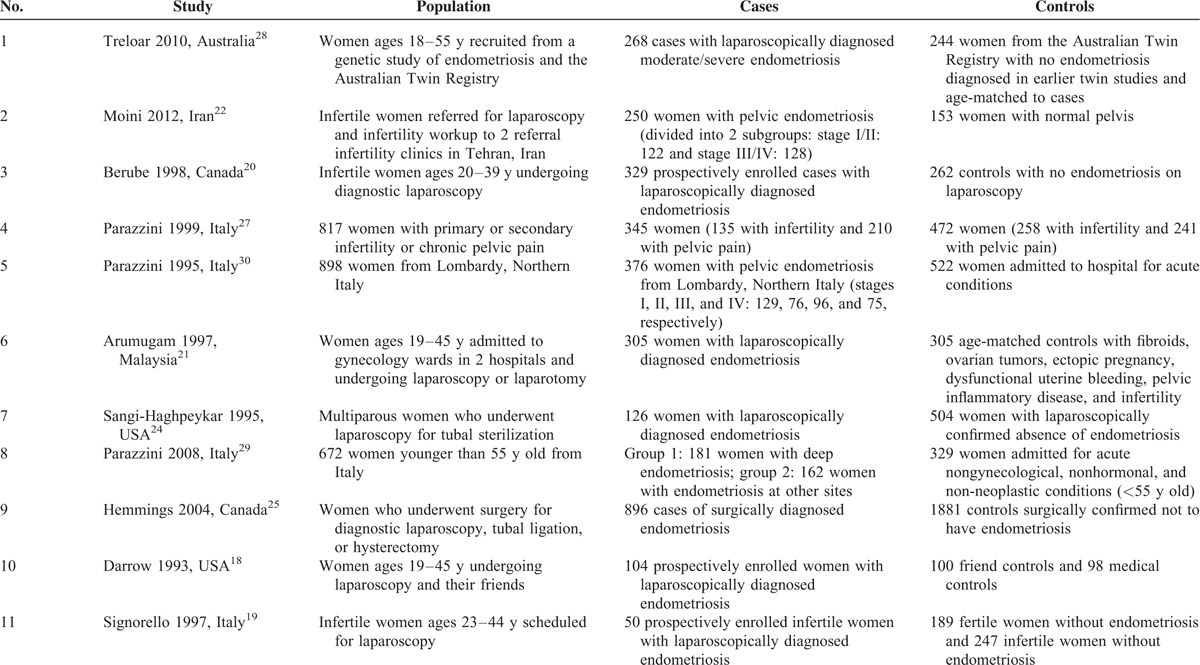
Characteristics of the 11 Case–Control Studies Included in the Analysis

### Data Quality

The quality of the studies was assessed by 2 investigators using the NOS, a validated tool for evaluating observational and nonrandomized studies, and the results were scored from 4 to 8. In particular, 1 study^19^ received a score of 8, 2^18,20^ a score of 7, 3^21,25,28^ a score of 6, 1^24^ a score of 5, and the remaining 4^22,27,29,30^ a score of 4 (Table 2).

**TABLE 2:**
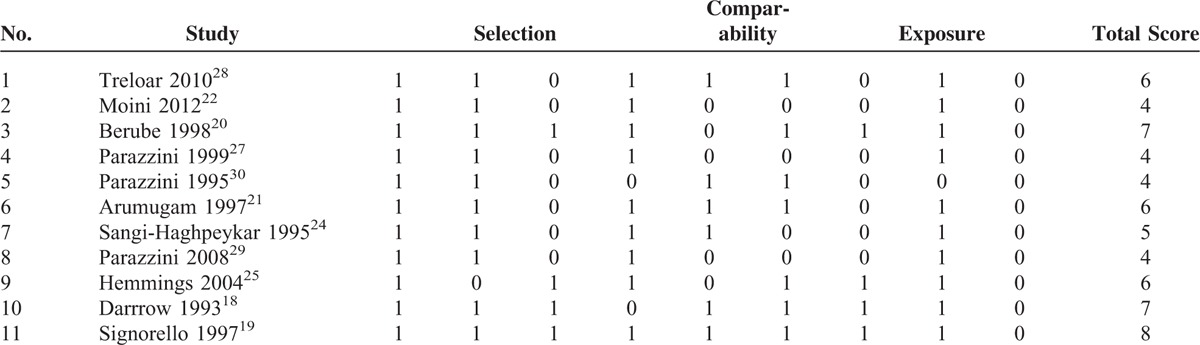
Quality of the Included Studies Assessed Using the Newcastle–Ottawa Scale

### Menstrual Cycle and Risk of Endometriosis

**FIGURE 2:**
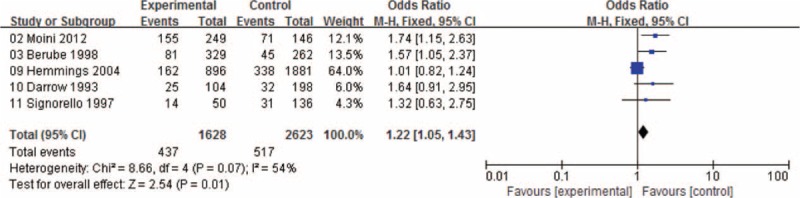
Forest plot of ORs with 95% CIs for menstrual cycle length SEQ27 and risk for endometriosis. CI = confidence interval, OR = odds ratio.

**FIGURE 3:**
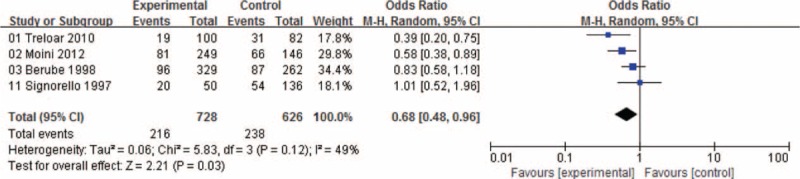
Forest plot of ORs with 95% CIs for menstrual cycle length LEQ29 and risk for endometriosis. CI = confidence interval, OR = odds ratio.

Women with menstrual cycle lengths SEQ27 were at an increased risk of developing endometriosis (OR: 1.22, 95% CI: 1.05–1.43; Figure 2) and those with menstrual cycle lengths LEQ29 were at an approximately 32% lower risk of endometriosis (OR: 0.68, 95% CI: 0.48–0.96; Figure 3).

### Heterogeneity Analysis and Sensitivity Analysis

There was heterogeneity among studies in overall comparisons. We assessed all of the comparison models by country, and study sample size (≤300 subjects or >300 subjects) to explore sources of heterogeneity across studies. However, none of these variables could explain the heterogeneity. In the sensitivity analysis, we repeat the meta-analysis by omitting each study 1 by 1 to exam the influence of each study on the pooled OR. The result proved that our results were reliable. In addition, when excluding 1 study^24^ simultaneously, the I^2^ significantly decreased from 79% to 49% which was not so high as before. Therefore, the study^24^ was considered as the source of heterogeneity and finally excluded when analyzed.

### Publication Bias

**FIGURE 4:**
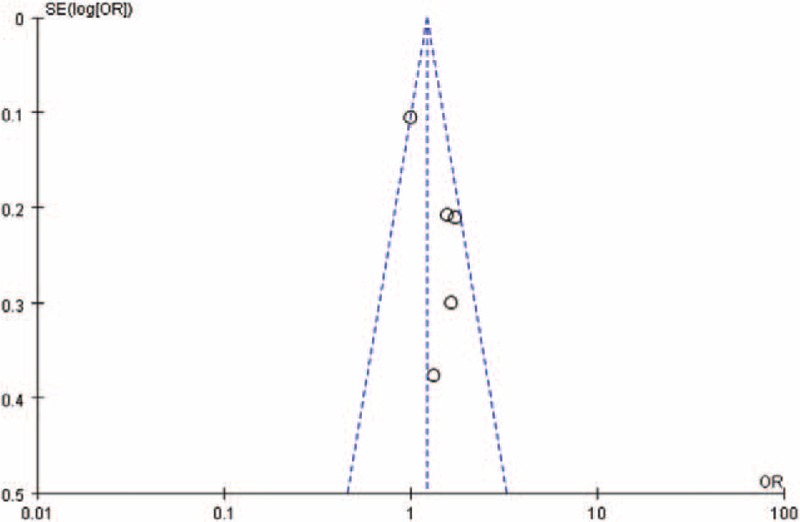
Funnel plot for the association between menstrual cycle length SEQ27 and risk for endometriosis.

**FIGURE 5:**
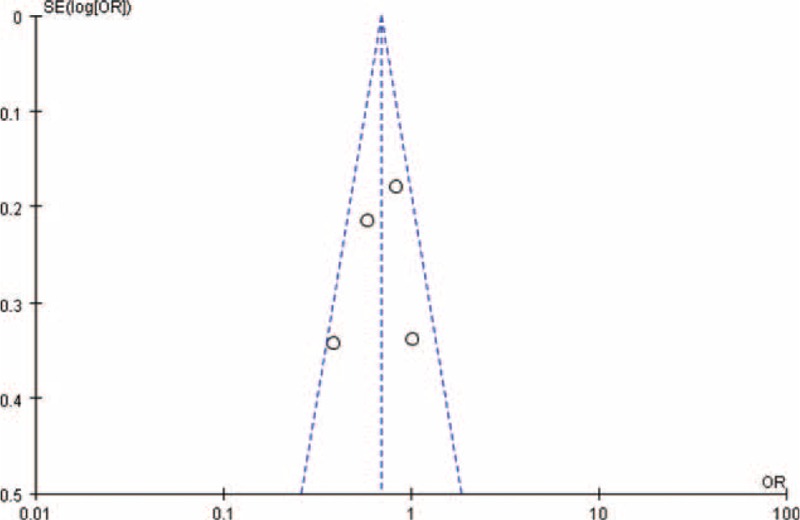
Funnel plot for the association between menstrual cycle length LEQ29 and risk for endometriosis.

Funnel plots for the association between menstrual cycle length SEQ27 or menstrual cycle length LEQ29 and risk for endometriosis were shown in the Figures 4 and 5. Visual inspection of funnel plot asymmetry was conducted to assess the potential publication bias. Obviously, no indication of asymmetry was observed in the funnel plots.

## DISCUSSION

The consistency of the results of this meta-analysis indicates that menstrual cycle length SEQ27 is inversely associated with the risk of endometriosis and menstrual cycle length LEQ29 decreases the risk.

The retrograde bleeding hypothesis can be 1 explanation for these findings. Sampson's theory of retrograde menstruation,^31^ which is supported by the anatomical distribution of endometriotic implants^32^ and higher prevalence of the disease in women with obstructive Müllerian anomalies, is currently considered to describe the main causal mechanism of endometriosis.^15,33^ A short menstrual cycle usually means an increased menstrual frequency and higher risk of exposure to menstruation. Menstrual cycle length SEQ27, which is shorter than average cycle length, could potentially increase the frequency and the risk of retrograde bleeding and finally increase the incidence of endometriosis. However, the cycle length LEQ29 influences the endometriosis in a contrary way.

The other potential explanation for our study results may be the fact that endometrial-like tissues of short-cycle-length (SEQ27) women are at a relatively higher frequency of exposure to higher levels of estrogen than that of long-cycle-length women. Several studies have already found that the ectopic growth of endometrial-like tissue was depended upon sex steroid hormones^34^ and local growth factors^35^ such as insulin-like growth factor 1 (IGF-1)^36^ or vascular endothelial growth factor (VEGF).^37^ Commonly, short cycle length comes along with punctually ovulation and the estrogen level sharply increasing in a short period of time before ovulation. Estrogen has synergistic effects with IGF-1/VEGF and sometimes enhance the effects of IGF-1/VEGF on ectopic endometrial cells proliferation and mitosis,^38^ which possibly ended with endometriosis.

Consistent with our conclusion, some studies^20–23^ concluded that short menstrual cycle length increases the risk of endometriosis. Oppositely, Sangi-Haghpeykar's study^24^ draw a conclusion that long cycle length is a risk factor. The opposite conclusion may result from the differences in the subjects’ source population, race, geographical region, and age. In summary, findings of our study can be used to guide the diagnostic and therapeutic strategies of endometriosis in clinical practices. For instance, the artificial menstrual cycle can be tried to manage the development of endometriosis in terms of delaying the menstruation or ovulatory phase. There are some limitations in our studies: only studies published in English between 1993 and 2014 were included; and the number of included studies and the total sample size were relatively small. More well-designed studies should be conducted.

## CONCLUSIONS

In conclusion, this meta-analysis suggests that menstrual cycle length SEQ27 increase the risk of endometriosis and length LEQ29 decrease the risk.
